# When the Nose Doesn’t Know: Canine Olfactory Function Associated With Health, Management, and Potential Links to Microbiota

**DOI:** 10.3389/fvets.2018.00056

**Published:** 2018-03-29

**Authors:** Eileen K. Jenkins, Mallory T. DeChant, Erin B. Perry

**Affiliations:** ^1^First Year Graduate Veterinary Education Program, Public Health Activity - Fort Bragg, United States Army, Fort Bragg, NC, United States; ^2^Department of Animal Science, Food & Nutrition, College of Agricultural Science, Southern Illinois University, Carbondale, IL, United States

**Keywords:** working canine, canine management, canine olfaction, canine performance, canine microbiota

## Abstract

The impact of health, management, and microbiota on olfactory function in canines has not been examined in review. The most important characteristic of the detection canine is its sense of smell. Olfactory receptors are primarily located on the ethmoturbinates of the nasal cavity. The vomeronasal organ is an additional site of odor detection that detects chemical signals that stimulate behavioral and/or physiological changes. Recent advances in the genetics of olfaction suggest that genetic changes, along with the unique anatomy and airflow of the canine nose, are responsible for the macrosmia of the species. Inflammation, alterations in blood flow and hydration, and systemic diseases alter olfaction and may impact working efficiency of detection canines. The scientific literature contains abundant information on the potential impact of pharmaceuticals on olfaction in humans, but only steroids, antibiotics, and anesthetic agents have been studied in the canine. Physical stressors including exercise, lack of conditioning, and high ambient temperature impact olfaction directly or indirectly in the canine. Dietary fat content, amount of food per meal, and timing of meals have been demonstrated to impact olfaction in mice and dogs. Gastrointestinal (GI) microbiota likely impacts olfaction via bidirectional communication between the GI tract and brain, and the microbiota is impacted by exercise, diet, and stress. The objective of this literature review is to discuss the specific effects of health, management, and microbiota shifts on olfactory performance in working canines.

## Introduction

The extraordinary olfactory capability of the canine has long been used by humans for odor identification and discrimination (1). The canine’s capacity for odor detection has been reported to be as much as 10,000–100,000 times that of the average human, and the canine lower limit of detectability for volatile organic compounds is one part per trillion (ppt) (2). This heightened sense gives canines the ability to detect a vast number of chemical compounds containing molecules that display subtle differences in stereoisomeric structures (3). This sensitivity, the unique capability to detect a target odor among a myriad of odors in an operational environment (4), and the ability of the dog to learn by operant conditioning (5) has made the working canine an intrinsic component of law enforcement, military, search and rescue, medical and assistance/service functions worldwide. However, despite the critical nature of the service that our canine partners provide, evidence related to olfaction health and performance is underrepresented in the scientific literature. The objective of this review is to discuss the effects of management decisions related to diet and physical conditioning, medical care, and microbiota shifts on olfaction performance in working canines.

## Health and Disease

### Anatomy of Olfaction

To properly manage the health of the detection dog, one must understand the anatomy and physiology associated with olfaction. The major components of the olfactory system are the nasal cavity, olfactory epithelium and receptors, the vomeronasal organ (VNO), and the olfactory bulb. The nasal cavity is comprised of two chambers separated by the nasal septum, which are highly vascularized, primarily supplied by the sphenopalatine artery. Each nasal cavity chamber contains three turbinates (nasoturbinate, maxilloturbinate, and ethmoturbinate) (6) that contribute to increased mucosal surface area. However, total mucosal surface area may be heavily influenced by muzzle size and shape in the canine (7). Nasal turbinates project from the lateral chamber walls and contain a network of tortuous veins. Medial and dorsal to the turbinates is the olfactory cleft, where 5–15% of inhaled air is diverted, and multiple cranial nerves terminate. As inhalation occurs, air first reaches the maxilloturbinate where there are a small number of olfactory sensory neurons. Air continues to flow into the ethmoturbinates and paranasal sinuses and is then directed toward the pharynx (6). Engorgement of turbinates alters airflow into the olfactory cleft, affecting olfaction. Turbinate engorgement is reduced by exercise, hypercapnia, and increased sympathetic tone, whereas it is increased by cold air, chemical irritants, hypocapnia, and increased parasympathetic tone. Some airborne odorants/chemicals can stimulate trigeminal free nerve endings in the nasal mucosa, which cause sensations like warmth, coolness, sharpness, but not odor (8). The detection of odor occurs only through the olfactory epithelium and olfactory nerves.

The olfactory epithelium is comprised of neurepithelium lining the cribriform plate, dorsal septum, dorsal and middle turbinates, and pseudostratified columnar epithelium, with millions of olfactory receptor (OR) cells (ORC). Olfactory epithelium also contains supporting sustentacular cells that regulate the composition of nasal mucous, serve as insulators between ORCs, and protect the epithelium from damage from inhaled agents (9). The mucous layer of the nasal mucosa is derived from Bowman’s glands embedded in the olfactory epithelium; this mucous layer maintains normal nasal humidity levels and traps odorants (10). Normal olfactory perception depends on this moist receptor area (9).

Olfactory receptor cells project directly to the olfactory bulb, with axons terminating in the glomeruli of the olfactory bulb (11). The ORCs have cilia that have surface odor receptors; human ORC have approximately 25 cilia per ORC, but dogs have hundreds of cilia per ORC, permitting the detection of significantly smaller concentrations of odorants in canines. There are more than 220 million ORs in the canine nasal cavity, which allow a vast number of odorants to bind (12). There is only one type of OR per ORC, and odor intensity is proportional to the number of ORC activated; ORC also have receptors for hormones and neurotransmitters. Olfactory neurons only live for 30–60 days, but unlike other mammalian sensory cells, ORCs constantly regenerate (13). The number and type of ORCs present in an individual dog are dictated by breed, genetics and training (7, 14–17); this concept will be explored later in the manuscript.

Embedded in the membrane of ORC cilia are extracellular portions which bind odorant, and intracellular portions coupled to G-protein. When an odorant binds the extracellular portion of the receptor, the G-protein A-subunit breaks away, activating adenyl cyclase, which subsequently converts ATP to cAMP. cAMP amplifies the incoming signal from the odorant by activating multiple sodium gated channels (11). The two-step opening of gated sodium channels causes depolarization, and the resultant action potential is transmitted through the olfactory bulb. Each odorant is recognized by a unique combination of activated ORs (18). The ability of the detection dog to properly recognize odors relies on this function.

The VNO lies along the ventrorostral aspect of the nasal septum, is bilaterally symmetrical, and acts as an additional site of odor detection (19). The VNO sensory neurons detect chemical signals that stimulate behavioral and/or physiological changes (20), provides alternate neuronal pathway to the hypothalamus, and is very slow to adapt to odors. The VNO contains both receptor epithelium and non-receptor epithelium, which differ structurally in the types of nerve fibers and types of embedded cells (21). The VNO functions in the detection of non-volatile odorants, especially pheromones, and is believed to play a role in social behavior and reproduction (21).

**Figure 1:**
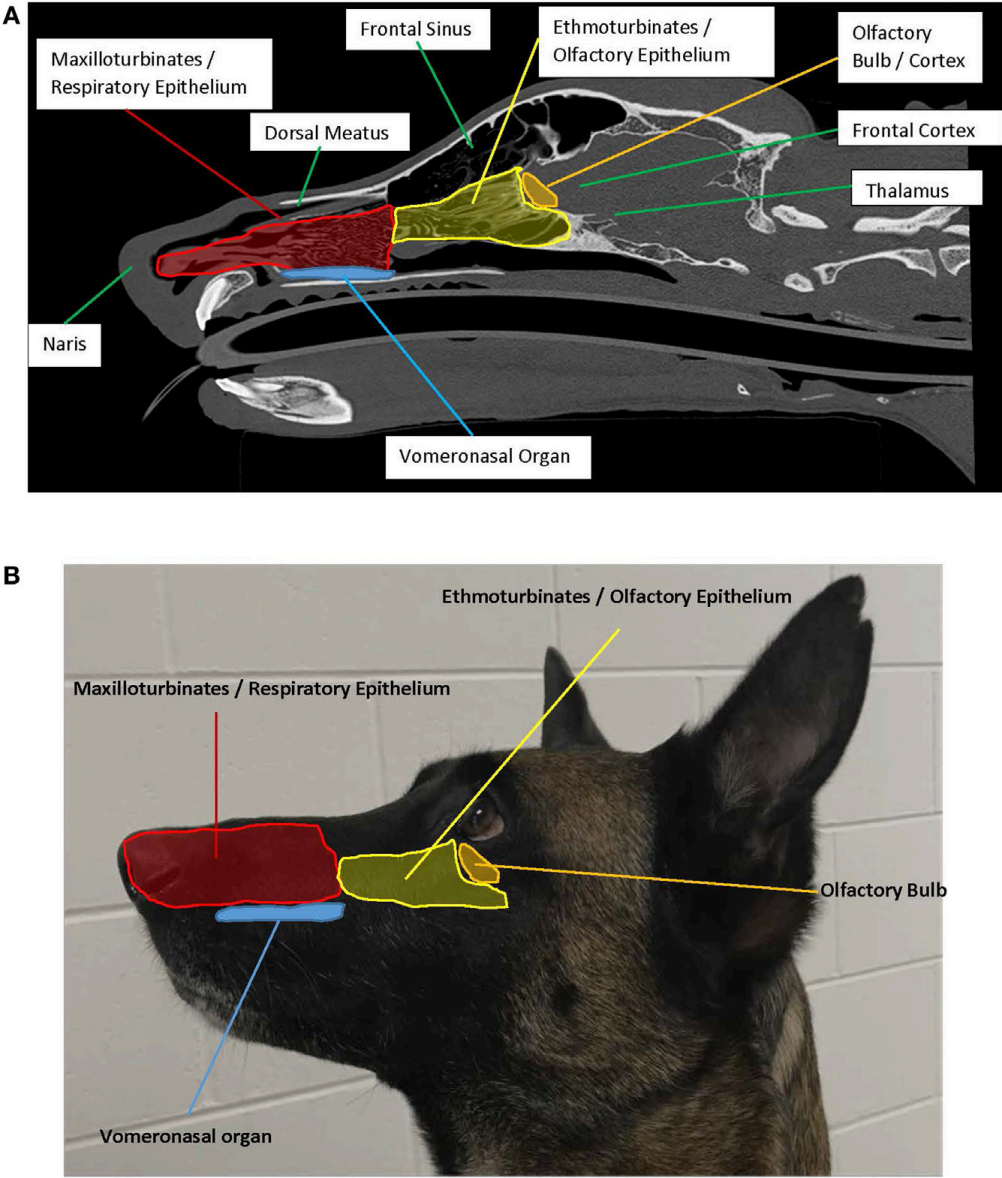
**(A)** Left sagittal plane highlighting the anatomy associated with olfaction. Photo credit: Adrien-Maxence Hespel, University of Tennessee. **(B)** Left exterior view demonstrating placement of interior structures associated with olfaction.

**Figure 2:**
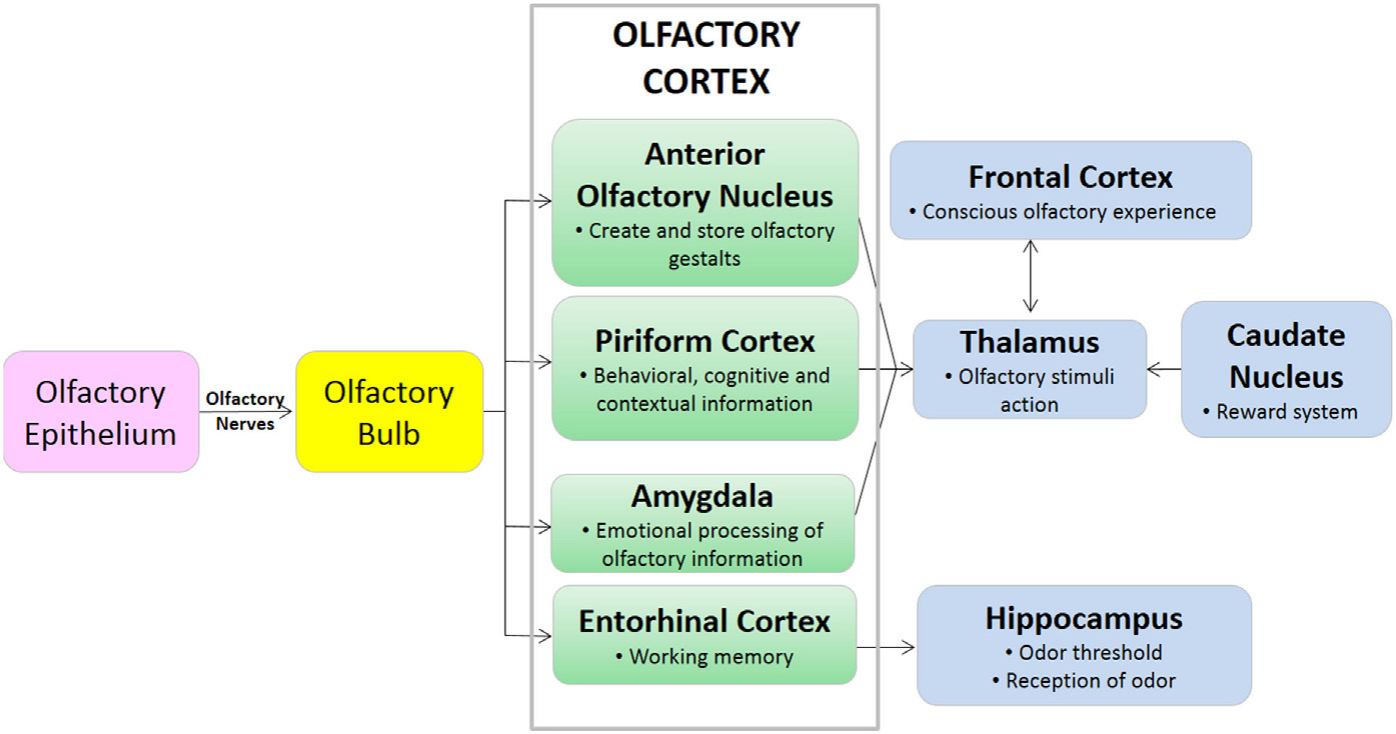
Diagram of pathway demonstrating olfactory signaling process.

The olfactory bulb is a paired structure, which functions primarily as a relay station, and to filter sensory input (6). There are approximately 1,000 ORC axons per second-order neuron, resulting in significant amplification of the odor signal. The mitral cells of the olfactory bulb project one primary dendrite to one glomerulus, and one axon to the olfactory cortex. The olfactory bulb is located under the frontal lobes, above the cribriform plate in humans, but is located more rostrally in other mammals, which may play a role in improved smell in lower mammals (19). The olfactory cortex is located within the medial temporal lobes and communicates directly with cerebral cortex. The olfactory cortex functions to receive sensory input from the olfactory bulb, permit conscious awareness of odor, identification of odor, odor memory, and odor localization in lower mammals. The olfactory bulb has both a sensory role (initial processing of olfactory information) and a modulatory role in the forebrain, hypothalamus, and limbic system (22). The olfactory pathway of canines is demonstrated in Figures 1 and 2.

The olfactory cerebral areas of the brain are divided into two functional categories: the neocortical (e.g., orbitofrontal complex) which regulates conscious odor perception, and the limbic (23). The limbic system is a collection of brain structures that collectively regulate olfaction, memory, behavior, and motivation. Components of the limbic system include the olfactory bulb, hippocampus, amygdala, and entorhinal complex, among others. The size and function of the limbic system varies across mammalian species, but in all species the limbic system has olfactory and non-olfactory components (24). The isocortex of the brain regulates higher-order functions such as sensory perception and cognition. While primates including humans have an inverse relationship between isocortex and limbic system volume, terrestrial carnivores including canines have high relative volumes of both the isocortex and limbic systems (24). These anatomical differences in brain component volumes may be partially responsible for the differences in olfactory capability between humans and canines.

### Physiology of Olfaction

Compared to humans, dogs have significantly larger surface area of olfactory epithelium, with approximately 30% more ORs that can recognize a much larger variety of odorants. Dogs also have the capability for excellent odor localization, even in presence of significant background odor, likely due to the larger nasal cavity size as compared to other species (25) and the unique airflow patterns created by sniffing (26). The ability to find the source of the scent, even in the presence of competing odors, makes the detection dog a critical partner in many military, law enforcement, and search and rescue operations.

**Figure 3:**
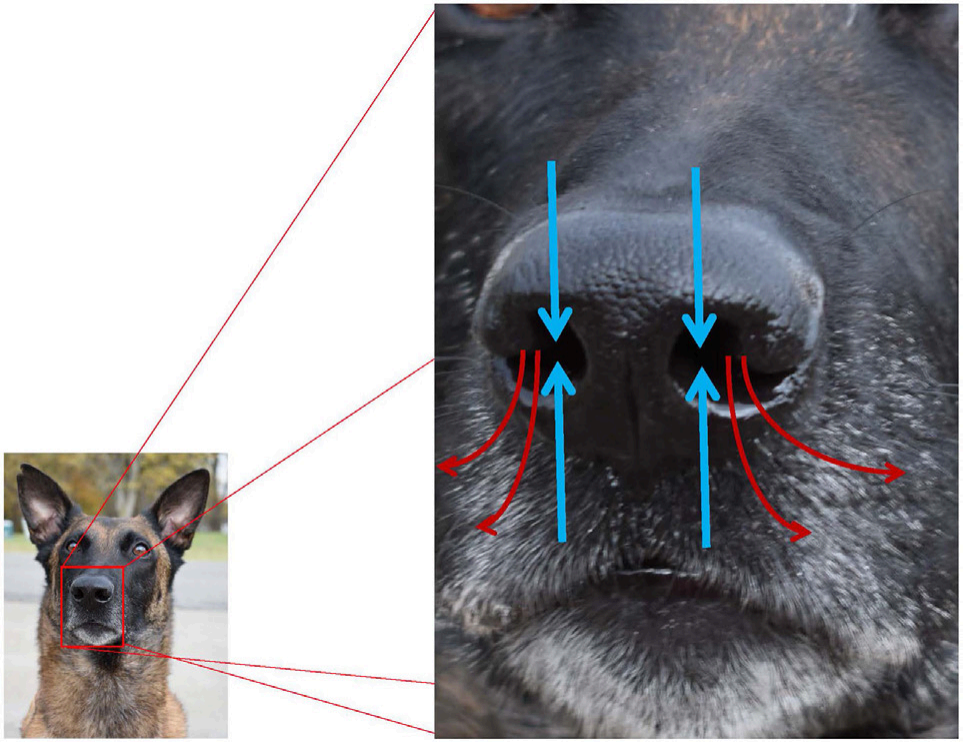
Pathways for inhalation and exhalation and airflow associated with olfaction (blue arrows = inhaled; red arrows = exhaled). Canines preferentially use the right nostril to sniff conspecific arousal odors and novel odors, and the left nostril to sniff familiar odors, non-aversive stimuli, and heterospecific arousal odors.

**Figure 4:**
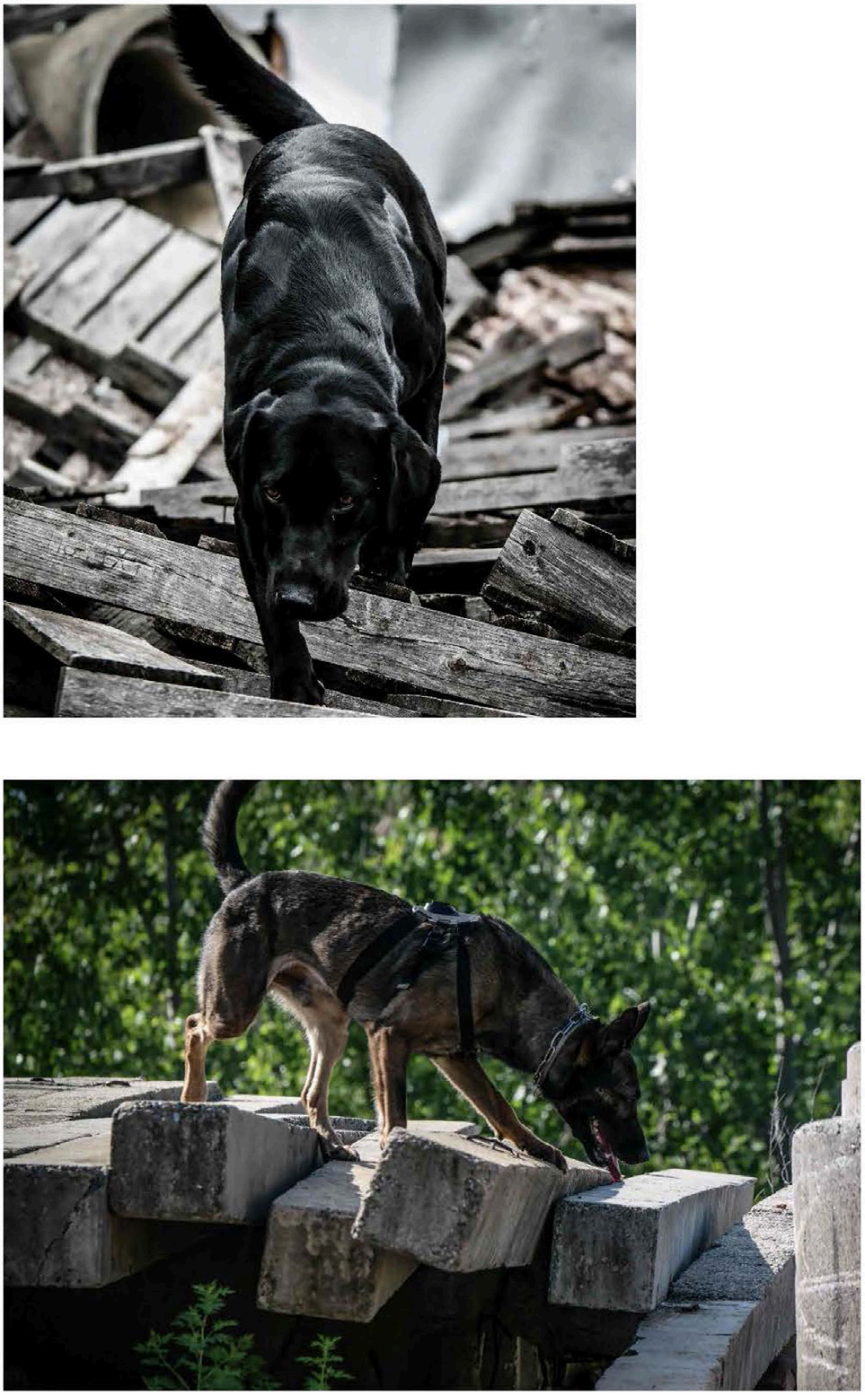
Disaster canines performing search work displaying the typical “nose down, tail up” posture associated with active olfaction. Photo credit to Tracy Darling.

During inspiration, 12–13% of air flow travels to the olfactory portion of the nose, and the remaining airflow is directed toward the nasopharynx where it exits the nasal cavity (26). Dogs have improved airflow sampling and odorant collection via active sniffing, which is the production of short, sharp breaths at 4–7 Hz, independent of canine body size (26). The average dog inhales 30 ml of air per nostril per sniff (19), and air is inhaled from the front and exhaled to the side as seen in Figure 3; this permits more efficient sampling of odorants. When a canine is sniffing, air within approximately 1 cm of the nostril is drawn toward the naris (26), and the high velocity air flow is transported to the dorsal nasal cavity where it turns 180° and flows back over the ethmoturbinates. Each nostril samples air separately, yielding bilateral odor samples that assist in odor source localization (26). In contrast to humans and other microsmotic species, air does not enter or exit the olfactory recess of the dog during expiration, resulting in prolonged exposure of inspired air to the chemoreceptors of the olfactory epithelium and continued olfactory stimulation throughout the respiratory cycle (26). For the working canine, active sniffing during “nose down, tail up” searching (see Figure 4) and efficient localization of odor source are critical to completion of the mission.

Environmental conditions, such as relative humidity and barometric pressure can have direct impacts on olfaction, in addition to the impacts those factors have on the generation and movement of odor itself. Philpott et al. (27) reported that olfactory thresholds in humans were independent of room temperature, peak humidity and peak inspiratory nasal flow. A subsequent, larger study reported by Kuehn et al. (28) subsequently determined that olfactory threshold level was impaired in hypobaric conditions, and olfactory thresholds were lower (sense of smell improved) in a humid environment. Search and rescue dogs perform better when relative humidity is high (29), potentially due to improved nasal humidity and odorant trapping. Humidity, but not rain, increased the efficiency of dogs in tracking and searching tasks by increasing odor intensity (30), and improved olfactory detection of pheromones, leading to increased mating activity during monsoon season (31).

Sniffing is advantageous compared to normal inhalation because it produces unidirectional laminar flow to the dorsal meatus and sensory epithelium of the ethmoturbinates (26, 32), increases the sensitivity to odors (32), drives activity in the olfactory cortex, and affects odorant intensity and identification (33). Nasal airflow patterns as described by Craven et al. (26) enhance olfactory acuity in the dog, but do not fully explain macrosmia, the enhanced ability to smell, in the canine. Lawson et al. (34) described the transport of specific types of odorants and the subsequent impact on olfaction. Odorant deposition patterns correspond to the anatomical organization of OR neurons: highly soluble odorants are deposited in the front of the olfactory cleft (dorsal meatus and nasal septum), whereas moderately soluble or insoluble odorants are deposited throughout the entire olfactory cleft (34). This combination of anatomical organization of OR neurons and airflow patterns induced during sniffing are likely responsible for the macrosmia widely demonstrated in working canines. Canines move more slowly and the period of sniffing lasts three times longer during the deciding phase of olfactory tracking (the “find”), as compared to the initial search phase and tracking phases (35). Concha et al. (36) demonstrated that sniffing patterns in working canines can be used to differentiate true negative from false negative responses. Trained scent detection dogs spent significantly less time sniffing true negative samples (no odor; no alert response), with only one sniffing episode for true negative samples (36). For detection dogs, these sniffing characteristics may result in more efficient detection work during a lengthy work cycle.

Dogs have repeatedly demonstrated “hemispheric specialization,” that is hemisphere specific brain processing of emotional, acoustic, and olfactory stimuli (37, 38). Unlike other senses, olfactory pathways ascend from the point of detection (nasal cavity) to the point of perception in the brain (olfactory cortex) ipsilaterally: right nostril sensory input is delivered to the right brain hemisphere, and left nostril sensory input is delivered to the left hemisphere (37). Canines preferentially use the right nostril to sniff conspecific arousal odors and novel odors, delivering sensory input to the right brain hemisphere, which processes threatening and alarming stimuli. Canines preferentially use the left nostril to sniff familiar odors and non-aversive stimuli such as food, as well as heterospecific arousal odors (such as human fear-induced sweat samples) (37). D’Aniello et al. demonstrated that hemispheric specialization and chemosignaling enhances communication of emotional states (including stress) between dogs and humans (39). For detection dogs, this likely means that target odors are being processed through the left nostril.

Anatomical connections between the olfactory pathways of the amygdala and piriform cortex and the limbic system underlie the interconnection between olfaction and memory (9). Olfaction and other forms of learning/memory are regulated by the same neurobiological rules (40). In working canines, memory of smell is of critical importance: when does odor memory begin? How many odors can canines remember? How long do canines remember trained odors? How long can a dog maintain olfactory performance without training? Canines learn odor starting in the prenatal period, due to the influence of maternal diet on the composition of the amniotic fluid (41), but the learned odor memory appears to dissipate by 10 weeks of age (42). Olfaction and cognition have both been demonstrated to deteriorate with age in the canine, but no specific age exists at which the dog ceases to learn (40). Williams and Johnston (43) determined that canines could readily learn and subsequently identify 10 odors in a search task. Given that domestic canines have demonstrated the ability to learn and remember more than 200 words (44) and the names of more than 2,000 toys (45), it is likely that working canines can remember far more than 10 odors. The durability of memory on trained odors has not been extensively studied, but Johnston (46) demonstrated that in explosive detection canines there was no systematic deterioration in detection performance for up to 4 months. Training methods can impact durability of odor memory, or at least the signaling from canine to handler when a specific odor is detected. If alerts are not reinforced, or if the canine conducts several searches without detecting a trained odor, the alert or search behavior can be extinguished (47). It is unclear, however, if memory is maintained when alert or search behavior is extinguished or what the maximum duration of time is that a canine can maintain odor memory without training (47).

Genetics are increasingly recognized as a critical component of olfaction in canines, with a comprehensive review published elsewhere (7). There are four types of receptors involved with olfaction and chemosensation in the dog: OR, vomeronasal receptors, trace amine-associated receptors, and formyl peptide receptor-like proteins. Most research on the genetics canine olfaction has been focused on OR genes. The canine OR repertoire is composed of 1,094 genes, approximately three times more than a human. This large genetic repertoire is believed to be related to the macrosmia evident in canines, producing an expansive array of ORs that permit the detection of broad ranges of odorant (48). In the canine, approximately 20% of OR genes are functionally inactive pseudogenes, but the percentage of pseudogenes varies by breed, and is significantly lower than microsmotic species like humans, in which as much as 50% of olfactory genes are pseudogenes (49). Polymorphism of OR genes may also impact olfactory capability and sensitivity in breeds and individuals. Tacher et al. (15) reported that both the percentage of pseudogenes and the frequency of specific gene polymorphism varied by breed, and speculated that genetic changes may contribute to differences in olfactory capabilities between breeds and individuals. This may offer some insight into the “working lines” within some breeds that tend to produce higher frequencies of successful detection canines than others.

The current literature contains conflicting information about breed-specific olfactory capacity. Jezierski et al. (50) demonstrated that German Shepherds were significantly better at detecting narcotics than Labradors and Terriers. In contrast, Hall et al. (5) reported that Pugs consistently outperformed German Shepherds and Greyhounds in olfactory acquisition and discrimination tasks. Polgar et al. (51) reported that “scent-group” dogs (e.g., basset hound, German pointer, etc.) performed better on a natural detection task than “non-scent” dogs (e.g., English greyhound, Afghan hound, etc.) and “short-nosed” dogs (e.g., Cavalier King Charles spaniel, Boston terrier, etc.). Additional research is needed to determine if breed specific olfactory capabilities are correlated with genetic polymorphism or if olfactory performance is more a function of behavioral attributes like inherent motivation (i.e., drive) and trainability.

### Hyposmia: Disease and Medication

Hyposmia, defined as decreased sensation of smell, is characterized as type I, II, or III. Type I hyposmia is the inability to recognize odorants correctly. Type II hyposmia is a quantitative decrease in the ability to recognize odorants, recognized in working canines as change in threshold or persistent failure to alert to previously trained odorants. Type III hyposmia is a decrease in estimation of the magnitude of odors; this type of hyposmia is only recognizable in humans. The causes of hyposmia can be broadly categorized as conductive disorders, sensory losses, or neural causes (52). Conductive hyposmia results from the failure of odorants to reach the olfactory mucosa, e.g., nasal inflammation, excess mucous production, and physical obstruction by space-occupying masses (polyps, neoplasia, etc.) (53). Sensory hyposmia is caused by damage to the olfactory mucosa, e.g., viruses, toxic chemical or industrial agent exposure, and neoplasia (54–56). Neural hyposmia is caused by lesions of the central or peripheral nervous system, specifically the olfactory cortex, olfactory bulb, and cranial nerves I (olfactory) and V (trigeminal), e.g., head injury (57).

### Disease

The impact of disease on olfaction has been widely documented in human medicine. In fact, “degradation in the sense of smell is a sentinel condition, particularly for neurodegenerative diseases such as Alzheimer’s” (58). Conditions associated with hyposmia or anosmia in humans include congenital disorders (e.g., Kallaman’s syndrome), endocrine or metabolic disorders, infections, inflammation, neurologic disorders including head trauma, local processes, occupational exposure to dust and toxic chemicals and materials, advanced age, and uremia (8, 57, 59–61). Hyposmia and anosmia are frequently self-reported in human medicine, but self-reporting may overrepresent the actual prevalence of hyposmia in humans. The prevalence of hyposmia in canines is unknown, but hyposmia in detection canines could be catastrophic.

When a canine is exposed to prolonged, increased body temperatures due to environmental or exertional extremes without the ability to acclimate properly, thermal injury may occur (62). Thermoregulation is compromised during heat stress; the canine increases respiratory rate (i.e., panting) and heart rate to compensate and cool the body. Panting decreases olfactory efficiency in canines and may lead to relevant hyposmia. Exogenous factors that may contribute to thermal stress and increase the likelihood of compromising olfaction include lack of acclimation to a novel environment, elevated humidity, lack of access to water, and poor ventilation (63). Heat injury likely leads to olfactory compromise, but the magnitude and duration of altered olfaction is unknown; further study is indicated.

As in humans, disease may impair olfaction in canines. Although there is limited canine research available, viral infection with canine distemper (64) and canine parainfluenza virus infections (65) have caused alterations in olfaction. Canine parainfluenza virus increased nasal inflammation and mucous secretions, causing a conductive hyposmia by reducing the contact between odorants and olfactory or trigeminal receptors in the nasal cavity. In addition, nasal inflammation, like that caused by canine distemper or parainfluenza viruses lead to vascular congestion in the respiratory mucosa, altering air flow patterns in the nasal cavity (65). Endocrine disease (e.g., hyperadrenocorticism, diabetes mellitus, and hypothyroidism) and neurologic disease (e.g., granulomatous meningoencephalitis and nasal tumors) have also been reported to cause hyposmia in canines (55); the exact mechanism of hyposmia in these disease states is not known but is likely neural. Recently, vomeronasalitis was associated with intraspecific aggression in cats (66). Asproni theorized that the inflammation present in the VNO and nasal cavity impaired sensory epithelium function and intraspecific communication but did not examine olfactory function in the studied cats. Given our understanding of the VNO and nasal physiology, it is likely that vomeronasalitis causes both sensory hyposmia and disrupted intraspecific communication in cats, and possibly in dogs. Trauma is a well-documented cause of neural hyposmia in people, but the impact of head trauma on olfaction in dogs has not yet been studied. If a detection canine experiences head trauma associated with lack of consciousness, evaluation of olfaction is indicated (67). Olfactory function diminishes with age in humans through a variety of mechanisms including altered nasal engorgement, cumulative damage to the olfactory epithelium, decreased mucosal enzymes, loss of selectivity of ORCs and neurodegenerative disease (68). Similar age-related changes were found in the olfactory system of dogs older than 14 years and were prominent in dogs over the age of 17 years (69). The older dogs had a decrease in number of ORCs and the number of cilia on ORCs. Interestingly, the older dogs demonstrated senile brain changes such as cerebrovascular amyloidosis in the olfactory bulb, but not in the olfactory mucosa. Disease-induced, but not age-induced, hyposmia in humans is generally reversible, possibly because olfactory neurons regenerate readily, but the duration of hyposmia and normalization of function cannot be predicted (54, 68); this is also likely true in canines.

### Pharmaceuticals

Type II hyposmia is common in humans during or after pharmaceutical therapy (70); the hyposmia is usually bilateral and temporary. The list of pharmaceuticals known to induce hyposmia in humans is long, including: anesthetics, antiarrhythmics, antihistamines, antimicrobials, antiproliferative and immunosuppressive drugs, endocrine drugs, gastrointestinal (GI) drugs, neurologic drugs, and NSAIDs (8, 57, 59). Pharmaceuticals frequently cause hyposmia through impairment of odorant binding to the OR or injury to the OR (sensory hyposmia), or through neurologic impairment (neural hyposmia).

Most relevant information on pharmaceuticals impacting canine olfaction is extrapolated from human medicine. Zinc metabolism is directly related to olfaction function in both humans and laboratory animals. Zinc nanoparticles, when added to explosives, enhanced the odorant response in trained canines in a dose-dependent manner (70). Zinc chelation, however, causes sensory hyposmia at the OR level. Some cardiovascular drugs such as angiotensin-converting enzyme inhibitors (ACE-I) (e.g., captopril) chelate zinc and cause hyposmia in humans (8); this effect has not been studied in canines. Anesthetics are documented to cause hyposmia in humans; the impact on olfaction in canines is presently being researched at Auburn University.

Antimicrobials such as metronidazole and doxycycline are commonly prescribed to working canines to treat diarrhea and vector-borne diseases, respectively. Metronidazole has been reported to cause hyposmia in humans (8) and to decrease olfaction performance in detection canines (71). Doxycycline has been reported to cause hyposmia in humans (60) but does not cause hyposmia in detection canines (71). Jenkins et al. noted that 50% of trained explosive detection dogs demonstrated an elevation in olfaction threshold when administered high-dose metronidazole for 10 days, but doxycycline administration at standard doses for 10 days did not impact olfaction. Metronidazole-induced hyposmia could not be predicted based on male or female sex, neuter status, or age but hyposmia was temporary, as olfaction threshold returned to normal within 10 days of discontinuation of metronidazole. Alternative medical interventions should be considered when appropriate prior to the use of metronidazole for detection dogs; if metronidazole must be used, it should be used at the lowest efficacious dose for the shortest duration possible.

Steroids can cause hyposmia in humans (8) and in canines (72). Ezeh administered high doses of dexamethasone or hydrocortisone combined with deoxycorticosterone to laboratory dogs and noted hyposmia without apparent clinical signs after 7 and 18 days of treatment, respectively. The noted steroid-induced hyposmia in dogs was attributed to elevation in the olfactory detection threshold. However, studies of humans with nasal inflammation demonstrated that the administration of oral and/or intranasal steroids sometimes improved olfaction, likely due to the resolution of nasal inflammation (73–75). Thus, veterinarians and canine handlers should carefully weigh the clinical need for steroids against the potential effects on olfaction. The mechanisms of pharmaceutical-induced hyposmia include impairment of odorant binding through altered mucus quantity or quality (e.g., antihistamines), inhibition of normal turnover/regeneration of olfactory neurons (e.g., steroids and chemotherapeutics), nasal vasoconstriction (e.g., decongestants), enzyme-associated effects of drugs (ACE-I), altered levels of cyclic GMP (phosphodiesterase blockers), and zinc chelation (cardiac medications) (76).

Given the paucity of research on pharmaceutical-induced hyposmia in canines, handlers, trainers and veterinarians caring for detection dogs should exercise caution with pharmaceuticals known to cause hyposmia in humans. It is also important to consider which medications may be biotransformed by the GI microbiota when discussing medical care for working canines. Information on reduction, hydrolytic and other chemical reactions for commonly prescribed medications and their associated impacts on microbiota and olfaction should be considered. Olfaction threshold and discrimination ability should be tested in any detection dog that has been treated with hyposmia-inducing pharmaceuticals prior to return to work.

## Management

There is a myriad of factors that can improve or compromise the performance of working canines. Frequency, intensity, and duration of work cycles should be considered prior to making management decisions particularly as pertains to olfactory acuity. Detection dogs (explosives, narcotics, search, and rescue) are different than sport dogs (agility, hunting, sled) and are measured with very different performance criteria (see Table 1).

**Table 1:** Categories of working canines and typical disciplines associated with each.

Sport	Detection	Service
Nose work^a^	Explosives^a^	Guide
Field trial/hunt test^a^	Narcotics^a^	Hearing
Agility	Search and rescue^a^	Mobility assistance
Flyball	Medical^a^ (cancer, research)	Emotional support
Rally	Pest^a^	PTSD
Barn hunt^a^	Arson^a^	Allergen detection^a^
Sled dogs	Conservation^a^	Medical^a^ (diabetes, seizure)
Obedience^a^	Invasive species^a^	Therapy
Conformation	Agriculture^a^	
Dock jumping	Patrol/apprehension^a^	
Lure coursing	Currency^a^	
Protection sports^a^	Prison (mobile phone)^a^	
Rally	Tracking/trailing^a^	
Herding sports	Firearm^a^	
Tracking^a^		
Weight pulling		

Factors to consider in the management of working canines		

Duration	Length of work cycle—# of hours spent performing work Example: agility course takes minutes to complete vs. guide dog working during all waking hours	

Frequency	Incidence of work—# of times called to perform work Example: daily missions (law enforcement) vs. “on call as needed” (disaster)	

Intensity	Energy exerted performing work—this should include physical as well as mental energy needed complete assigned task Example: patrol dog released to apprehend suspect vs. border patrol dog screening vehicles as they move through checkpoint	

*^a^An olfactory component associated with job function*.

Conditions that can alter a dog’s working potential include breeding and selection, regular fitness and conditioning, and the development of a dietary regimen that meets the nutrient requirements and utilizes quality ingredients. Maximizing olfactory function should be paramount in decisions regarding detection dogs. A summary of selected publications associated with working canine performance is presented in Table 2.

**Table 2:** Summary of selected studies reporting effects on olfaction/performance associated with management or medical care.

Citation	Treatment	Classification	Duration	Olfaction response
(77)	Exercise and fat supplement	*n* = 18 Hunting	12 weeks	Coconut oil decreased olfactory acuity in non-conditioned dogsExercise decreased olfactory acuity in non-conditioned dogs

(78)	Exercise and fat supplement	*n* = 17 Explosive detection	12 weeks	Corn oil increased olfactory acuityExercise decreased olfactory acuity

(79)	Quail hunting and dietary protein	*n* = 23 Hunting	11 months	Animal-based protein increased olfactory acuity

(80)	Hunting and dietary fatty acids	*n* = 23 Hunting	12 months	EPA, DPA, DHA increased olfactory acuity

(72)	Steroids	*n* = 24; companion	28 days	Dexamethasone or hydrocortisone + DOCA decreased olfactory acuity

(32)	Exercise and panting	*n* = 6 Explosive detection	20 min treadmill	Olfaction and panting display inverse relationship

(47)	Conditioned odorant	*n* = 10 Companion	Odor condition 7 days	Conditioned odorant increased olfaction sensitivity

(81)	Handler–canine interaction	*n* = 60 Companion	3 months	No handler influence

(71)	Metronidazole	*n* = 18 Explosive detection	10 days	Degradation of detection threshold for 9 canines

(50)	Odor detection scenarios; novel environment; training	*n* = 164 Narcotics detection	Unknown	Final stage of training decrease olfactory acuityKnown and novel environment similar olfactory acuity

(82)	Scent detection (live find and human remains)	*n* = 11 live find *n* = 12 cross-trained	Unknown	Cross trained canines compromised on alerting live scent when cadaver scent present

(1)	High intensity training	*n* = 13 Shepherd breeds	5 days per week; 18–20 months	High olfaction sensitivity and specificity

(65)	Canine parainfluenza virus (CPI virus)	*n* = 10 Companion	3 weeks	CPI virus prevented contact of odoriferous substances with olfactory receptors

(83)	Helicopter travel	*n* = 9 FEMA search and rescue	30 min helicopter travel	No effect on search performance or gut microbiota

(84)	Novel and known odorants	*n* = 21 Explosive detection	6 weeks	Decreased target performance with no exposure prior to scenario

(85)	Commercial air travel	*n* = 6 FEMA search and rescue	2.5 h air travel	No effect on search performance in spite of change to gut microbiota and fecal scores

(86)	Handler–canine interaction	*n* = 5 Military	10 days	Elevated handler anxiety improved canine target detection

### Conditioning and Training

As one might expect, training and physiological conditioning significantly impact olfactory performance. Decreased find rates using certified detection dogs on scent wheels have been reported following exercise (78); this is likely explained by the increased panting that typically occurs following exercise. Canines that were physically conditioned maintained greater olfactory acuity compared to canines that were not physically conditioned when both groups were challenged with exercise. Non-conditioned canines displayed a 63.6% decrease in olfactory sensitivity following exercise (78). Physically conditioned canines have a lower exercising heart rate compared to their non-conditioned counterparts and this improved cardiovascular condition may contribute to better thermoregulatory performance and subsequently decrease the need for panting (32). Other supporting work has shown that a rigorous training program leads to high frequencies of correct target alerts (32). Immediately following extreme physical exercise, there is a reduction in the sniffing rate and increased panting rate which result in reduced olfaction performance (32). This may be explained by the fact that non-conditioned canines pant harder during intense exercise instead of breathing through their nose, which decreases the quantity of odorants passing over olfactory epithelium in the nasal cavity (77). It seems clear that physical conditioning (specifically as pertains to minimizing panting) may support improved olfaction in the detection dog.

Scent detection training techniques can also directly impact olfaction. Wang et al. (16) and Youngentob and Kent (17) demonstrated that dogs develop more ORs for odorants on which they are regularly trained. Gerritsen and Hank (14) also reported that ORC cell turnover is not static: new replacement ORC type is triggered by familiar scents. Simple odorants and complex odorants induce different neural responses in scent detection dogs. Wilson and Stevenson (87) theorized that cortical synaptic plasticity is enhanced by experience with odorants (simple or complex) in a variety of conditions. Gerritsen and Hank (14) further suggested that dogs will learn complex odors more rapidly if they are first trained on individual components of the odor, but results vary across studies. Fischer-Tenhagen et al. (88) found that detection dogs trained with mixtures of odor containing the target odor had more correct indications when the target odor was tested in a new context, than dogs trained on a pure reference odor. These data provide scientific evidence for the traditional training concept of “proofing” detection dogs with the use of distraction items. Functional MRI of the olfactory system in trained scent dogs indicated that odor concentration impacts brain activation: low odor concentration resulted in unilateral brain activation, whereas high odor concentration resulted in bilateral brain activation (58). In addition to odor type and frequency, training techniques impact olfaction sensitivity and discrimination. Pavlovian conditioning significantly improved odor acquisition (89) and improved resistance to disruptors (90). Continuous reward systems worked best for acquiring a behavior such as learning to discriminate a specific odor, and intermittent rewards worked best for maintaining a learned behavior (40). More research is needed to determine the impact of training simple versus complex odor, the impact of odor concentration on learning, and the interaction of genetics and training on performance in detection dogs.

### Hydration

Management of the detection dog in the field may often involve mitigation of dehydration and fatigue. Dehydration of the nasal mucosal membrane results in decreased enzyme activity and decreased membrane fluidity, altering neurosignal transduction and odorant receptor function. A combination of decreased airflow and dehydration of the mucosal layer can significantly decrease odor detection capabilities in the working canine (77). Dehydration in search-and-rescue canines was reported to occur in dogs working after the terrorist attacks on 9/11 (91, 92), the Haiti earthquake (93), and the Washington landslide (94). One recent study examined three intervention strategies for hydration of canines (95). Border patrol vehicle inspection canines were utilized (high frequency, low intensity searches) to investigate the benefits of water, oral electrolyte solution, or subcutaneous fluids for rehydration of canines working in hot conditions. The authors reported no clear benefits for any of the strategies examined but did note that voluntary consumption of the flavored oral electrolyte solution was higher as compared to water alone. Increased voluntary fluid consumption contributed to improved hydration. No benefits associated with the use of subcutaneous fluids were noted. On the contrary, hydration with subcutaneous fluids was associated with an increase in creatinine that was noted to indicate either dehydration or potential muscle damage. No information on dietary regimens was provided by the authors and behaviors recorded were not affected by hydration strategy. Olfaction as a measure of performance could not be quantified; standardized olfaction testing was not possible because of the operational nature of the field study. This study demonstrates that dehydration in the field is a concern which warrants more investigation especially when considered in relation to potential olfactory challenges.

Thermal recovery was enhanced when using a low protein diet top dressed with corn oil in Labradors exercised on treadmills (96). The authors reported lower core body temperatures 10 and 20 min following exercise and lower rectal temperatures in dogs fed a maintenance diet topped with corn oil as compared to dogs consuming the performance ration without corn oil. Olfaction acuity was not measured in this study. Conversely, hunting find rates in English Pointers improved in dogs fed a higher protein, higher fat (31:21%) diet, as compared to a diet containing lower protein and fat (26:17%) (79). Thermal recovery was not investigated. Factors associated with fatigue were not reported in either study. Extrapolation across studies is challenging due to the difference in methods, ingredients and parameters measured but thermal recovery and olfactory impact should be weighed heavily in decisions regarding diets for detection dogs.

### Nutrient Content

The nutritional requirements for canine athletes have previously been examined in review (97). Mullis et al. (98) examined the maintenance energy requirements specific to detection dogs and reported that they were approximately twice the known resting energy requirement (RER = 70 kcal × BW^0.75 kg^). The authors noted no differences in energy requirements across breed, age, or gender, but did report a significant effect associated with number of searches performed. This is particularly interesting because the work performed by these dogs simply required that they be active and alert; it was not reported as physically strenuous. Findings in these dogs suggest that there may be an unexplained energy requirement associated with the mental focus/attention required by working canines. Duration, frequency, and intensity of work likely all impact energy requirements for the working canine. The impact of surgical sterilization on olfaction is unknown, but spaying of racing Greyhound bitches produced no change in overall performance, motivation, or racing speed (99).

Exercise and diet seem to be inextricably linked to canine performance, but there are few studies examining the relationship between these elements of detection dog management. English Pointers withheld from exercise and fed a diet supplemented with coconut oil appeared to experience compromised olfaction, but exercised dogs maintained olfactory acuity (77). The authors reported greater olfactory sensitivity for all exercised dogs regardless of dietary fat source (beef tallow; beef tallow + corn oil; beef tallow + coconut oil). Angle et al. (78) demonstrated benefits to olfaction when using corn oil supplemented diets and exercise.

The improved olfaction observed with increased polyunsaturated fatty acid (PUFA) content in the diet has also been reported in rodent studies (100). Rodent studies have also been used to measure olfactory sensitivity associated with nutritional status and have reported improved olfaction associated with fasting (101, 102) and compromised olfaction as a result of satiety (101, 103). These findings are believed to be linked to the appetite inducing hormone ghrelin, which contributes to exploratory and sniffing behavior and improves olfactory sensitivity (104). This critically important work demonstrates a potential link between fasting and improved performance in the detection dogs. Anecdotal reports from seasoned trainers have often included recommendations for letting the dogs work hungry; these data may provide evidence for this long-standing canine training technique. Food has been documented to be a more effective reward than praise or petting but has not been compared for effectiveness against toys (105). Hall et al. (90) reported inconsistent responses for dogs offered presession feeding when odor discrimination tests were conducted. For detection disciplines requiring dogs to work independent of the handler (disaster, explosives), use of fasting to improve exploratory and sniffing behavior may be a useful training tool to examine. Further study is needed to determine the appropriate diet titration to maximize olfaction, the length of fasting time necessary, and the potential impacts on olfaction performance.

### Diet and Behavior

The relationship between diet and behavior has been well studied in other species, but few studies have examined the relationship between diet and behavior in canines (106). Docosahexaenoic acid (DHA) is necessary for optimal neurological development in puppies, and lower concentrations of DHA have been associated with aggression in German Shepherd dogs (40). PUFAs are essential to membrane function and control of oxidative stress, especially in the hippocampus of the brain, the area responsible for associative learning (40). Hennessy et al. (107) reported a reduction in adrenocorticotropic hormone upon exposure to novel stimulus for those fed a premium (44% animal-based protein) diet as compared to those fed a maintenance diet (17% animal-based protein). Other studies have shown a reduction in territorial aggression in client-owned dogs fed a lower protein diet (106–109). DeNapoli et al. (110) reported that low protein diets with supplementary tryptophan reduced aggression in dogs. Sechi et al. (111) utilized a dietary intervention strategy of nutraceutical supplementation (including tryptophan) in dogs with behavior disorders. They reported a subsequent increase in serotonin, dopamine, and β-endorphins indicating reduced aggression, and reduced plasma cortisol and noradrenaline indicating reduced markers of stress. These studies offer a glimpse into the potential application of dietary manipulation for stress and aggression management. The need for working canines to operate without aggression in stressful environments warrants further research in this area. However, reduction of dietary protein could be a dangerous undertaking and this topic would require extensive research prior to the use of this mitigation strategy for dogs in the field.

## Microbiota

### Understanding the Microbes

The GI microbial community is a complex ecosystem containing bacteria, fungi, archea, and protozoa. Improvements in molecular techniques such as next generation sequencing have increased our study and subsequently our understanding of both the composition and function of the GI microflora. However, there remains a great many unanswered questions regarding the impacts associated with changes in the microbiota on the overall health and performance of the working canine.

As more studies are published highlighting changes in the GI microbiota, it is increasingly important to understand how those changes are measured and how that data is presented (112–115). Microflora, microbiota, and microbiome are all words that seem to permeate the discussion in many scientific communities. Microflora is a term that refers to the collective community (fungi, archaea, protozoa, bacteria) in question. Bacteria are referred to as the “microbiota.” Studies referencing the term microbiome are generally describing the genome of the microbiota and typically include information about by-products of fermentation (VFA’s, pH, etc.) as well as genetic information about the community constituents (116).

Microbiota studies are typically visually presented to answer taxonomy-related questions such as (1) How many and which microbial communities are present? (2) What is the diversity of the population? Taxonomic diversity is generally represented using alpha and beta diversity. Alpha diversity (diversity within a given sample) is typically represented as a rarefaction curve and describes evenness and richness of a given sample (117). Rare microbial species are more likely to be missing from small samples, therefore, richness is an important factor to consider for small data sets. Alternatively, beta diversity (diversity between samples) is used to measure taxonomic similarity based on phylogenetic distance (118). Beta diversity also provides a visual assessment of the abundance (weighted) or presence (unweighted) of given taxa and is represented using a PCoA plot. Other techniques for visual depiction of data include heat maps or hierarchical cluster analysis.

Although a comprehensive discussion on the procedures associated with microbial sequencing is beyond the scope of this work, it is important to understand that primer selection and target region of the 16s RNA gene are critical (119, 120) and can cause significant variation in the results and subsequent interpretation of data generated. These techniques are culture-independent and have allowed researchers to greatly improve our understanding of GI microbiology. Data are highly impacted by several factors including sequencing techniques, primers, selection of correct hypervariable region and others. Inconsistent approaches used in many studies published have made it extremely difficult to make comparisons across data sets and continue to challenge interpretation.

Traditional culture-dependent techniques (i.e., Sanger sequencing) have allowed researchers to investigate the presence of specific pathogens and are useful to identify species commonly associated with GI disease such as *Salmonella, Campylobacter jejuni*, or *Clostridium perfringens*. However, these techniques are limited in their applicability as compared to currently molecular methods (i.e., next generation sequencing) that make taxonomic identification and metagenomics applications easier (121). The comprehensive characterization and community identification required for microbial profiling of the GI tract requires the more sensitive techniques associated with next generation sequencing and has become the accepted standard for microbial studies.

### Microbial Balance

The GI microbial ecosystem harbors significantly different communities within each compartment (122, 123). Predominant phyla in working canines are similar to other monogastric species and are typically dominated by Firmicutes and Bacteroidetes. The characterization of the collective GI microbial community and associated function is beyond the scope of this work and has been previously reported elsewhere (114, 122, 123). Resident bacterial groups within the GI tract play an intrinsic part in the regulation of homeostasis; their role in the regulation of the host innate immunity has been well described (124–126). The microbiota comprises part of the intestinal lumen barrier, contributing to the protection of the GI ecosystem via competition for nutrients and adhesion sites and by secreting compounds thought to inhibit the colonization of non-resident microbes (127). This may explain why puppies are generally more at risk for GI disease associated with pathogens such as *C. jejuni* as their bacterial profile may not yet be fully mature enough to provide sufficient protection or deterrence (128).

Microbial community structure variation between individuals is consistently present (129). Age, breed, and gender have all been shown to affect the microbial profile across multiple species (130–132). Cohabitation of humans and dogs has also been shown to impact the microbial community (133). The authors concluded that the factors affecting microbial homeostasis are not the same for the oral and GI communities as compared to the skin community. These data suggest that GI changes are related to other, heretofore, unknown factors. These reported variations must be considered when evaluating published microbial data. Studies including dogs across several age groups, breeds, and with both genders should be analyzed accordingly to account for the variation associated with those factors.

### Microbial Imbalance

While it is relatively easy to predict the factors that will affect the microbiota (age, gender, breed, antibiotic use, travel), it is slightly more difficult to predict the associated impacts to the dog. Current evidence suggests that alterations in the GI microbial community can fundamentally alter the structure and function of the GI lumen; this has been termed “leaky gut syndrome” with prior review elsewhere (134). This condition describes the physical changes to the intestinal lumen associated with changes in the microbial profile and is particularly concerning because of the potential for immunological disruption and bacterial translocation resulting in endotoxemia. By-products of healthy microbial fermentation, specifically short chain fatty acids (SCFA’s), are thought to provide energy for the host and contribute to the mediation between the microbial ecosystem and activation of the immune system (135).

High levels of bacterial diversity are generally associated with good health; diminishing diversity has consistently been reported with negative health outcomes in humans such as obesity, diabetes, and GI disease (136). Reductions in the phyla Firmicutes and Bacteroidetes, which are typically dominant, along with concomitant increases in Proteobacteria have been reported in dogs diagnosed with chronic GI inflammatory disease (137). Minamoto et al. (138) demonstrated slightly different microbial impacts but that may be due to the variation inherent with different techniques, breeds and ages of dogs sampled. Development of a dysbiosis index (DI) has offered a diagnostic tool to categorize microbial data into a simple ratio reflecting normal microbiota (DI < 0) or microbiota indicative of chronic enteropathies (DI > 0) (139). Unfortunately, the use of this index requires laboratory testing and is limited by its very small initial data set. However, the concept provides an important step in the direction of assessing fecal samples diagnostically with recommendations for treatment and dietary interventions.

The bidirectional communication that occurs between the brain and gut (microbiota–gut–brain axis) provides some insight into the dysbiosis that has been reported as a result of environmental stress (140). Stress associated with travel, change in environment, and physical exertion are common in the working canine. Changes in the fecal microbiota of working canines following in-cabin transport via commercial airline resulted in an impact on both abundance and type of bacteria and were accompanied by a poorer fecal score (85). Conversely, when researchers examined the effects of helicopter travel stress in working canines the relatively short nature of the stressor (hot-loading and 30 min of flight) did not result in any effect on the microbiota (83). Notably, both studies reported no effect on performance as determined by total search time or previously identified stress behaviors. The duration and type of travel required to induce microbial dysbiosis has not been examined in working canines.

### Dietary Modification of the Microbiota

While studies in dogs are limited, some data have shown promising results for microbial manipulation through the use of different fiber supplements on microbial community and resulting SCFA production (112, 113, 132, 141–144).

Researchers examining the use of fructooligosaccharides reported improved production of butyrate, a volatile fatty acid beneficial to colonocyte and epithelial cell repair, as well as reductions in *C. perfringens*, a potentially pathogenic microbe. A second study yielded similar results along with increased numbers of bifidobacteria, a potentially beneficial microbe (144). Other work in sled dogs fed a synbiotic (combined pre- and probiotic) reported decreasing incidences of diarrhea (141). If researchers can develop dietary mitigation strategies that consistently reduce or prevent GI distress, this may benefit dogs working in field scenarios with limited access to veterinary intervention. The use of dietary supplements that may mitigate or prevent the onset of GI distress warrants further study.

Diet has long been identified as the dominant factor impacting microbial community structure (112, 113, 144–148). What we don’t know is what impact meal size and frequency has on the GI microbiota. Handlers frequently must adjust meal times and sizes for detection dogs throughout the course of a mission. Data in horses has demonstrated an effect on the GI microbiota associated with meal frequency and size (148); it is not known if a similar impact would be observed in the monogastric canine. Information elucidating potential impacts on the microbiota would be helpful in managing concerns associated with diarrhea in the field.

### Microflora and Olfaction

The densely populated microbial niche in the GI tract has been reported to play a key role in the regulation of behavior and brain function. The microbiota–gut–brain axis influences neurotransmission and behavior. It therefore might be the key in nutritional interventions for maintaining brain and olfaction health (149), with early microbial modulation resulting in long-term impacts on stress-related physiology and behavior (150). Given the relatively unexplored nature of the communication occurring between the gut microbiota and the stress response system of the brain, it seems reasonable to question whether alterations of the gut microbiota could play a role in stress reduction as evidenced by the display of stress behaviors in the dog.

The olfactory epithelium has been generally overlooked regarding the potential role of microorganisms on the development and efficiency of odorant transduction. ORs are formed by many G-protein coupled receptor proteins that identify volatile odorant molecules (151). Originally it was thought ORs were only located in the olfactory epithelium. In the GI tract, ORs have been identified in enterochromaffin cells; these receptors can affect the secretion of serotonin in response to fragrant molecules with subsequent effects on GI motility (152). Serotonin also plays a critical role for olfactory information processing as the olfactory bulb is comprised of serotonergic fibers and was recently shown to effectively regulate the flow of olfactory processing in mice (153). Given the link between GI microbiota and serotonin regulation, it seems likely that a relationship exists between the GI microbiota and odorant detection although as yet it is unknown (154).

Nasal microbiota community structure has been linked to olfactory function (155). Human subjects demonstrated differences in microbiota of people assessed for olfactory function with deficiencies related to the presence of butyric-acid producing microbes (155). These findings suggest that the microbial composition of the nasal passage can potentially shape or alter olfactory performance. The implications of altered olfactory performance associated with bacterial fluctuations in the nose are significant. The nasal microbiota of dogs with chronic rhinitis and nasal neoplasia was reported to differ in community structure when compared to healthy dogs (156). Isaiah et al. (157) identified an effect associated with job type on canine nasal microbiota. Even though all dogs were housed in a single facility and fed a single diet, researchers reported differences in alpha diversity for canines that was related to job type (vapor wake, patrol and narcotics, explosives). No differences were reported in beta diversity suggesting that species richness but not bacterial community structure was affected by the work done by dogs in each group (157).

One specific OR (OR51E1) has been detected in pigs along the entire GI tract from the gastric cardia to the rectum (152). OR51E1 colocalizes with an enteroendocrine cell marker all along the GI tract and was expressed in the greatest density in the duodenum. Duodenal enteroendocrine cells are the primary source of gastric inhibitory peptide and cholecystokinin. Duodenal enteroendocrine cells are equipped with multiple receptors connected to sweet and bitter tastes. OR51E1 gene expression in olfactory bulbs has demonstrated feedback mechanisms, differential activation of transcription factors, and epigenetic regulation. Circulating hormones that control food intake and energy balance modulate olfactory epithelium, and the ablation of olfactory sensory neurons in mice protected them from diet-induced obesity (158). There are several factors like age and diet that impact gut luminal microenvironment and the intestinal microbiota modulate OR51E1 gene expression in GI tract tissues (152).

## Future Directions and Unanswered Questions

We lack evidence-based data conducted in working canines that will allow us to fully investigate the links between microbiota shifts and any possible performance (i.e., olfaction related) or health sequalae. We know that diet can both change the microbiota and impact olfaction in other species. What we do not yet know is what mechanism (if any) exists that links olfaction with the microbiota. When one considers the unique microbial community harbored by the individual dog, does that explain why olfaction was only compromised in 50% of the dogs who were received metronidazole (71)? Is it possible that the reduction in Firmicutes experienced by dogs receiving metronidazole provides the key to the olfactory challenge they experienced (114)? If olfaction is enhanced as a result of fasting (102) and satiety reduces olfactory performance (103), should we be rethinking the timing of our feeding programs? What impacts will that fasting have on the microbiota of the working canine? The critical impact of the work conducted by these canines requires much deeper understanding of all things that could hinder their job performance. A more thorough investigation of factors associated with microbial changes and associated impacts on job performance (i.e., olfaction) is vital.

## Author Contributions

MD developed the concept and wrote the first draft of the manuscript. EJ and EP wrote major sections of the document in its current form. All the authors read, edited, and approved the final manuscript.

## Disclaimer

The views expressed herein are those of the author(s) and do not reflect the official policy of the US Army Medical Department, Department of the Army, Department of Defense, or the U.S. Government.

## Conflict of Interest Statement

This review was conducted in the absence of any commercial or financial relationships that could be construed as a potential conflict of interest.
